# Delayed Post-cholecystectomy Gallbladder Fossa Abscess Due to Citrobacter freundii

**DOI:** 10.7759/cureus.37169

**Published:** 2023-04-05

**Authors:** Simran Malhotra, Jacques Lara-Reyna, Eugenius J Harvey, Allen T Yu

**Affiliations:** 1 Department of Surgery, Icahn School of Medicine at Mount Sinai, New York, USA; 2 Department of Neurosurgery, University of Illinois College of Medicine Peoria, Peoria, USA

**Keywords:** gallbladder fossa abscess, gallbladder removal, citrobacter, abscess, cholecystectomy

## Abstract

While laparoscopic cholecystectomy has become the treatment of choice for cholecystitis, complications such as abscess development can result even years after the intervention. We present a case of a patient with a remote history of laparoscopic cholecystectomy now diagnosed with gallbladder fossa abscess infected with *Citrobacter freundii*, a low-virulence pathogen typically seen in iatrogenic urinary tract infections. Subsequent conjoint percutaneous drainage and long-term antibiotics resulted in both clinical and radiological improvement for the patient. Therefore, in the absence of recent events or risk factors for developing an abdominal wall abscess, a previous remote history of surgical intervention needs to be considered for the possible etiology, especially those with low incidences and long latency periods such as *Citrobacter*.

## Introduction

Since the 1980s, laparoscopic cholecystectomy has been a choice for the treatment of symptomatic cholelithiasis [1,2]. Despite its advancements, complications after laparoscopic cholecystectomy may include port insertion infection, intraoperative bleeding with possible conversion to open surgery, biliary duct injury, bile leak, retained stones, gallbladder rupture and spillage of stones in the abdominal cavity, and formation of an intra-abdominal abscess [1,3-7]. The complication rates of patients undergoing cholecystectomies range between 4.4% to 18.5% [3]. Abscess development in a post-cholecystectomy status is often linked to gallstone spillage [4,6-8]. The more common pathogens in these abscesses include gram-negative bacteria such as *Escherichia coli* and *Klebsiella sp.* [9-11]. Management and treatment for such include surgical drainage, appropriate antibiotic therapy, and gallstone removal if applicable [12].

*Citrobacter *is a facultative anaerobic, motile, Gram-negative bacillus most associated with iatrogenic urinary tract infections [13-15]. We present a case of gallbladder fossa abscess extending to the abdominal wall. After a detailed medical history, we did not find recent events for abscess development besides her remote history of laparoscopic cholecystectomy. The patient was ultimately managed on short-term drainage and six weeks of antibiotics with close follow-up for six months. The patient’s most current exam shows a fluid collection without evidence of an abscess.

## Case presentation

We present the case of a 64-year-old patient with a past medical history of hypertension, prediabetes, active *Helicobacter​*​​​​​​ (*H.) pylori* infection, and previous surgical history of laparoscopic cholecystectomy 20 years ago. The patient presented with progressive worsening of localized pain at the right upper quadrant (RUQ) with associated chills and subjective fevers for three weeks prior to admission. Six days prior to admission, the patient underwent outpatient upper gastrointestinal endoscopy and colonoscopy. She was diagnosed with *H. pylori *and started on two weeks of oral omeprazole (20 mg daily), oral clarithromycin (500 mg every 12 hours), and amoxicillin (500 mg x 2, twice a day). The patient denied alcohol or tobacco use, sexual activity, recent travel, or sick contacts at the time of admission.

On admission, vital signs showed a temperature of 38.1°C (100.6°F), heart rate of 65 beats per minute, respiratory rate of 18 beats per minute, blood pressure of 165/77 mmHg, and oxygen saturation at 97% on room air. Physical examination showed a palpable RUQ mass with tenderness but without skin changes. Initial blood work of significance is shown in Table 1.

**Table 1:** Initial patient blood work

Test		Result
White blood cells (K/mL)		14.3
White blood cell differential (%)	Neutrophils	80.1
	Lymphocytes	13.4
	Monocytes	6
	Eosinophils	0.4
	Basophils	0.1
Hemoglobin (g/dL)		10.6
Hematocrit (%)		30.2

**Figure 1:**
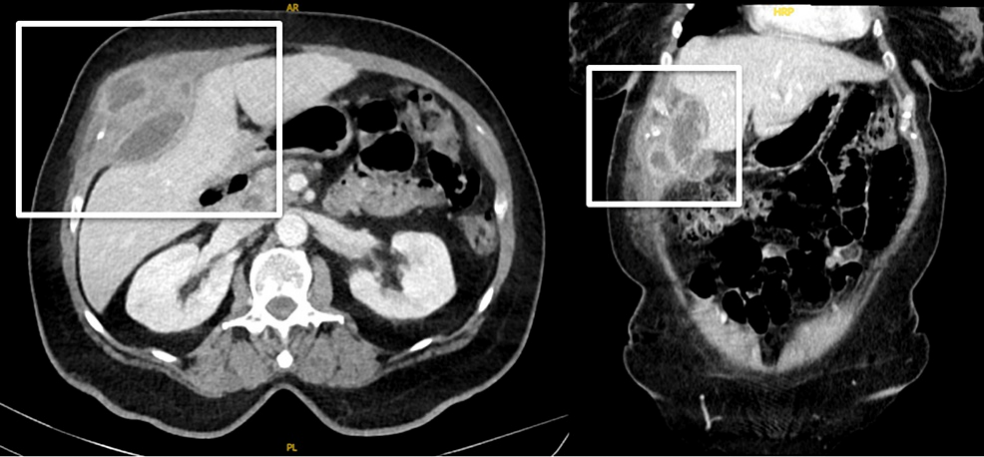
Multi-loculated anterior abdominal wall abscesses originating in the gallbladder fossa and causing mass effect on the anterior aspect of the right hepatic lobe

Abdominal computed tomography (CT) revealed a multi-loculated thick-walled fluid collection in the gallbladder fossa extending anteriorly, causing a mass effect on the right hepatic lobe, and measuring 5.4 x 2.5 x 5.0 cm (Figure 1).

**Figure 2:**
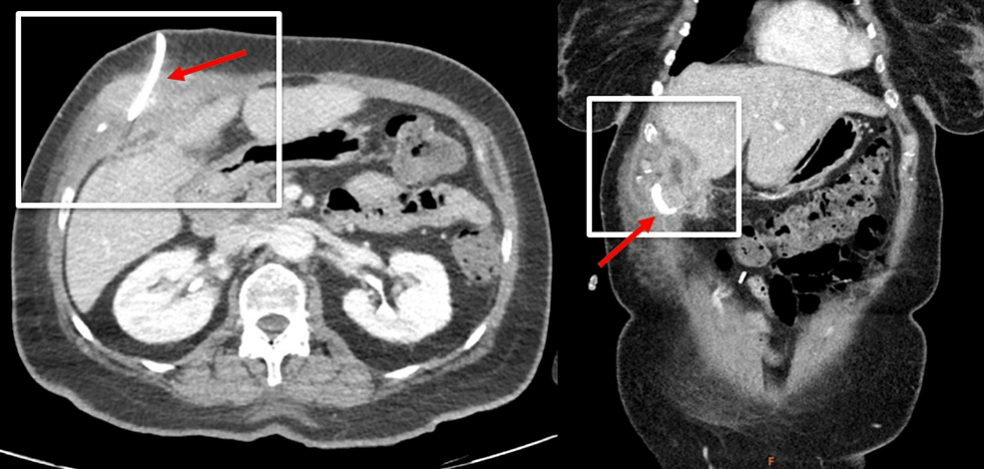
Post-drain placement follow-up CT showing interval decrease of the abdominal wall abscess Arrow: catheter in the center of the abscess

The patient was treated with IV ceftriaxone (2 g every 24 hours); oral metronidazole (500 mg every 8 hours); and continued on oral omeprazole (40 mg daily) and oral clarithromycin (500 mg every 12 hours). Interventional radiology performed image-guided drainage and drain placement, aspirating 15 cc of purulent fluid and placing an 8 Fr catheter into the abscess collection. The definitive aspirate culture was positive for *Citrobacter freundii*, identified by mass spectrometry, resistant to cefazolin (minimum inhibitory concentration of >16 μg/mL) and cefuroxime (minimum inhibitory concentration of 8 μg/mL). In the following days, the patient's drainage output was approximately 95 cc of fluid. Infectious disease was consulted and the patient was switched to ciprofloxacin HCl (500 mg orally every 12 hours) on hospital day six with a continuation of oral omeprazole (40 mg daily). The same day, a follow-up CT was performed, showing an interval decrease in size of the thick-walled collection now measuring 4.2 x 1.1 x 1.7 cm, which is an 88.2% volumetric reduction (Figure 2).

The patient was discharged on hospital day seven, given that her drain output was less than 10 cc over the last 48 hours in the absence of fever or worsening symptoms. She was discharged on oral ciprofloxacin (500 mg every 12 hours) for ongoing abscess treatment, oral tetracycline (250 mg every 6 hours), oral metronidazole (500 mg every 8 hours), and bismuth to complete her *H. pylori* treatment.

**Figure 3:**
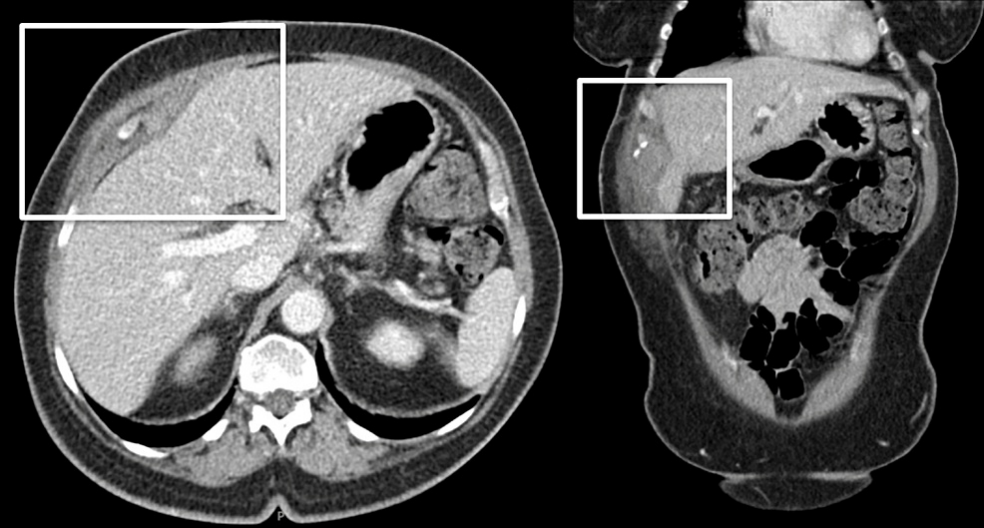
One-month follow-up CT showing interval improvement of the collection and removal of the catheter

Post-discharge, the patient was closely followed by infectious disease and surgery outpatient. One week after discharge, the patient’s drain was removed after approximately 12 days of its placement. One-month post-discharge, a follow-up CT revealed near resolution of the RUQ abdominal abscess, absence of fever or leukocytosis, and clinical improvement (Figure 3). At this time, the patient was continued on the ciprofloxacin regimen and metronidazole was added for two weeks. Upon follow-up with infectious diseases 38 days post-discharge, the patient had completed her course of ciprofloxacin and had two days left of her metronidazole regimen. A week later, the patient’s labs showed no leukocytosis and improved CRP of 7.05 mg/L, down-trended from the previous 30.87 mg/L, and the abdominal ultrasound showed 5.1 x 1.7 x 5.4 cm right upper anterior abdominal wall fluid collection with internal debris along the abdominal wall musculature, without evidence of abscess, now classified as seroma. Physical exam showed non-tender masses in the epigastrium and RUQ; both were decreased in size and softer to palpation.

Eight months post-discharge at a follow-up appointment with surgery, the patient reported minimal to resolved spontaneous drainage from the supra-umbilical collection, denying fever or pain. Palpable on physical exam, both the right subcostal seroma and the supra-umbilical collection had decreased in size.

## Discussion

Intra-abdominal abscess development is the most common complication due to gallstone spillage post-cholecystectomy [4,6-8,11]. This complication is more likely to occur during laparoscopic than during open cholecystectomy [6]. Demographically, of reported cases with a ten-year or greater latency, there is a female predominance of 89%, which is consistent with our patient [11]. The causative pathogens of these abscesses are often gram-negative bacteria, like *Escherichia coli* and *Klebsiella sp. *[9-11]. Management and treatment for such include surgical drainage, appropriate antibiotic therapy, and gallstone removal if applicable [12].

The scope of this report is focused on the development of intra-abdominal abscesses in patients undergoing cholecystectomy, which has been linked to intraoperative gallbladder rupture and stone spillage in multiple case reports [2,4,6]. However, in this case, there was no objective evidence from the patient or medical records regarding intraoperative gallstone spillage. We could only identify the previous cholecystectomy 20 years before the onset of the symptoms as the most significant factor associated with the abscess development. It should also be noted that spillage during a cholecystectomy should be properly noted in the operation report, and ideally, the patient should be informed.

While post-cholecystectomy infective complications have been reported in the literature, the chronicity of the latency period varies (ranging from one month to 20 years post-surgery across 28 cases with a peak incidence of four months), with the longest reported around 20 years after surgery, which is similar to our case [16]. Additionally, *Citrobacter *infections are rare; the genus is characterized as facultative, anaerobic, motile, Gram-negative bacillus, a low-virulence pathogen more commonly related to urinary tract infections in the hospital setting. In a long-term retrospective analysis of overall infections with this genus, a study reported only 78 isolated cases in an 11 years period, in which 73.2% of patients were related to urinary tract infections [13]. This genus belongs to the *Enterobacteriaceae *family, and the most frequently associated with active infection are *freundii *and *koseri *[8,13].

In studies published from 2000 to 2022, we found only two cases associated with *Citrobacter *among the reported post-cholecystectomy abdominal abscesses. The first case included a sub-phrenic abscess secondary to necrotizing pneumonia in the right lower lobe via trans-phrenic migration and its latency period was two years post-cholecystectomy. The treatment was drainage and antibiotics for eight weeks with satisfactory radiological and clinical improvement [17]. The second case was of an abdominal abscess that presented two years post-laparoscopic cholecystectomy, which was complicated by gallstone spillage and liver abscess drainage. It was treated with cefepime and then ertapenem upon discharge. Ten days post-discharge, the patient experienced worsening abdominal pain and a new CT scan showed the persistence of perihepatic fluid; the patient was then taken for surgical exploration and drainage. Cultures did not show bacterial growth, but he was discharged on ciprofloxacin plus metronidazole with abscess resolution [8]. Both cases associated with *Citrobacter were *shown to have a latency of two years post-cholecystectomy, which differs from our patient who has a remote history of 20 years.

*Citrobacter *abscess development in general is especially rare in adults, as it is more known to cause meningitis and brain abscesses in neonates [18]. In a review summarizing 12 cases of *Citrobacter* (*C.) koseri* abscesses unrelated to cholecystectomies, the common patient comorbidities were diabetes and pulmonary tuberculosis, and the common sites were in the brain and iliopsoas. Most cases were managed with drainage and antibiotics, namely, third- and fourth-generation cephalosporins, aminoglycosides, and fluoroquinolones. Hence, a similar treatment plan was used for our patient.

Overall, our report did not identify risk factors or recent surgery that explain the gallbladder fossa abscess development; the only potential risk factor was the cholecystectomy 20 years ago. The implementation of a minimally invasive approach by drain insertion into the abscess and the decision for long-term antibiotics driven by the results of cultures proved to be effective in this case. The importance of a multidisciplinary approach and proper medical management can be efficient in the setting of this unusual pathogen.

## Conclusions

*C. freundii* abdominal abscesses associated with previous cholecystectomies, though very rare, can be observed with a very long latency due to their low virulence and long doubling time. Conjoint percutaneous drainage and long-term antibiotics can result in clinical and radiological improvement. Our patient with the RUQ abscess and laparoscopic cholecystectomy history was managed with drainage and long-term antibiotic treatment of ciprofloxacin and metronidazole for two months, without the need for surgical exploration. Her abscess resolution was tracked through three follow-up CTs and one abdominal ultrasound over six months. Therefore, in the absence of recent events or risk factors for developing an abdominal abscess, a previous history of surgical intervention needs to be considered for the possible etiology, especially those with low incidences such as *Citrobacter*. Given the slow growth and latency of *Citrobacter*, long-term antibiotic treatment and close follow-up may be required for satisfactory resolution and to avoid the need for surgical intervention.
